# Reuse of bottom sediment from reservoirs to cropland is a promising agroecological practice that must be rationalized

**DOI:** 10.1038/s41598-025-92206-2

**Published:** 2025-03-04

**Authors:** Cécile Gomez, Julien Amelin, Guillaume Coulouma, Juliette Gaab, Subramanian Dharumarajan, Jean Riotte, Muddu Sekhar, Laurent Ruiz

**Affiliations:** 1 LISAH, Univ Montpellier, IRD, INRAE, Institut Agro, AgroParisTech, Montpellier, France; 2 Indo-French Cell for Water Sciences, Indian Institute of Science, ICWaR, Bangalore, India; 3 SAS, INRAE, Institut Agro, Rennes, France; 4 ICAR-National Bureau of Soil Survey and Land Use Planning, Hebbal, Bangalore, India; 5 GET, Univ.Paul-Sabatier, IRD, CNRS, Toulouse, France; 6 Civil Engineering Department, Indian Institute of Science, Bangalore, India; 7 G-EAU, Univ Montpellier, INRAE, AgroParisTech, IRD, Institut Agro, Cirad, Montpellier, France

**Keywords:** Circularity, Local knowledge, Soil properties, Sustainability, India, Agroecology, Sustainability

## Abstract

In semi-arid areas, intermittent streams are often equipped with small reservoirs to store water for irrigation and/or groundwater recharge, and to capture sediments lost through erosion. These reservoirs must be periodically desilted to maintain their storage capacity. While bottom sediments are generally considered waste, their reuse in agricultural fields is a centuries-old practice in India. Our study aimed to test the hypothesis that local farmers’ knowledge and current practices can help in understanding and rationalizing this practice. The study relied on both interviews of farmers and physico-chemical analysis of soil and sediment samples collected in a cultivated watershed in South India. First, our results disprove our hypothesis as we found a wide diversity of (i) application rates ranging from light soil amendment to creation of anthropogenic soils, and costs, which were not explained by the distance between reservoirs and fields neither by the field size, suggesting that there is no consensus among farmers on the optimal dose, and (ii) opinions on the impact of sediments on soil functions with the majority citing an improvement in the physical and/or chemical properties of the soil, suggesting that there is no consensus on the sediment impact on soil. Secondly, our results highlight that (i) only farmers with access to irrigation implemented this practice and they sourced sediment from the nearest reservoir, (ii) a slight majority of farmers used less irrigation water and less fertilizer after sediment application, and (iii) differences in sediments and soils composition suggest that sediment application is more likely to improve soil physical structure than nutrient status. The reuse of sediments on cropland could therefore be a promising agroecological practice, likely to increase the resource circularity and the sustainability of cropping systems. However, expressing its potential would require defining optimal application rates, assessing potential risks, sharing knowledge and promoting collective management of the resource.

**Supplementary Information:**

The online version contains supplementary material available at 10.1038/s41598-025-92206-2.

## Introduction

To face erratic rainfall, reservoirs have been built for centuries across the five continents for harvesting and storing surface water especially in semiarid regions^1^. Each reservoir is part of a structuring node of the agricultural landscape where water is stored during the rainy season and can be redistributed during the dry season^2^. In addition to irrigation, these reservoirs provide multiple services both for local population like drinking water, watering farm animals, fish production, washing, bathing and are also beneficial for the environment, by improving groundwater recharge, aquatic biodiversity and control of erosion by trapping sediment^3^. This latter service is crucial when intermittent heavy rainfall generates surface run-off and soil erosion, but it can also be seen as a disservice, as sediment deposition is responsible for siltation and subsequent drop of storage capacity^4^, as observed in many semi-arid regions such as in Zimbabwe^5^, Tunisia^6^, Italia^7^ and India^8^. Therefore, bottom sediment removal is essential to ensure reservoir sustainability in the long-term^9^.

The management of sediments removed from reservoirs is a worldwide issue^10^, as they are mostly seen as a waste rather than a resource, as highlighted by the European network SedNet^11^. The management options depend on bottom sediment physical and chemical characteristics^10^, but also on government policies and local stakeholder’s awareness. While the most common option is to recycle sediment for engineering purposes, including construction^10^, sediment reuse for agriculture is less common. Studies on sediment application on agricultural land are pretty rare and the conclusions about the benefits of this practice vary from one region to another. A study In Poland^12^ suggests that it increases soil pH value, soil organic C and N contents, plant-available P, K and Mg. In Brazil, Braga et al.^13^ claim that it can efficiently replace commercial chemical fertilizers. In Spain, Canet et al.^14^ report an improvement of soil physical properties (water holding capacity and cation exchange capacity). In India, few studies have reported an improvement of soil water holding capacity^15^, organic carbon or available nutrient status increase^16,17^.

In India the traditional “tank cascade system”, composed of series of small reservoirs along drainage networks, have been built over centuries not only to harvest water for irrigation but also to trap sediments and limit erosion losses at the catchment scale^8,18^. Across India, the current number of small reservoirs is estimated at around 0.3 million^18^, with 35% located in the South of the peninsula (Tamil Nadu, Karnataka and Andhra Pradesh)^19^. There, reservoir sediments are considered by most farmers as a valuable resource, and their application to agricultural land is an age-old traditional practice^20^ for filling eroded patches and enhance land productivity^[,8,21^. In the past, most farmers used their bullocks, unoccupied during the dry season, to transport sediment to their plots^22^, or even to their farm where it was mixed with manure before application to fields^20^. While the practice virtually disappeared in the 1980s^23^ with the decline in the use of bullocks in farming systems^20,22^, the recent development of motorization (excavators and tractors^24^) and of government-implemented programs of reservoir desiltation have given it a new momentum. However, to our knowledge, there exists no scientific basis to estimate the optimal input dose, according to soil and sediment properties, and to quantify the expected benefits for soil quality and crop production.

In this context, our study aims to test the hypothesis that local farmers’ knowledge and practices related to bottom sediment application in a cultivated experimental watershed in South India (Berambadi) can help to understand and rationalize this practice. To explore this hypothesis, we combined a survey based on interviews of farmers who spontaneously apply bottom sediment to their fields and a sampling campaign to characterize the variability of the physico-chemical composition of both sediments and soils.

## Material and method

### Study site

**Fig. 1:**
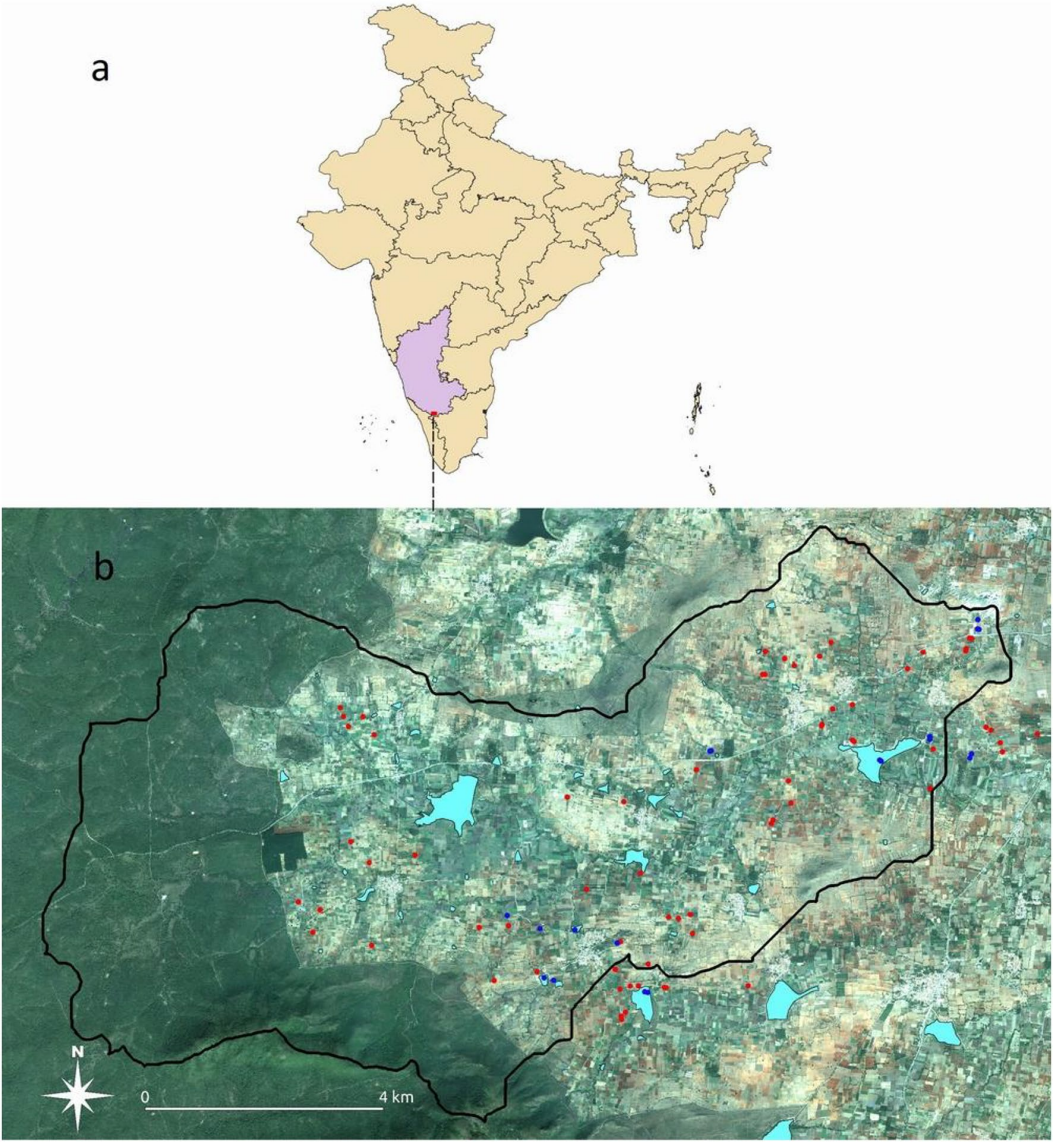
Location of the study site: (a) Karnataka state (purple color) location in India (b) map of the Berambadi watershed with the 66 surveyed plots (red dots) and the collected sediment and soil samples (blue dots), plotted over a PLEAIDES image acquired on 11-01-2020 (« Pléiades © CNES 2020, Distribution Airbus DS »). The watershed boundary is delimited with a black line and the reservoirs are coloured in cyan. The map was created by the authors using Qgis, version 3.10.4-A Coruña https://download.qgis.org/downloads/.

The experimental watershed of Berambadi (84 km^2^) is located on the Deccan Plateau in South India (Fig. 1a). It belongs to the Kabini Critical Zone Observatory (AMBHAS, SNO M-Tropics^25,26^, which is part of the OZCAR Research Infrastructure^27^. About 60% of the watershed is cultivated while the western part is covered by forest. The climate is tropical sub-humid to semi-arid with an average rainfall of 800 mm/year and a PET of 1100 mm (aridity index P/PET of 0.7). Three cropping seasons regulate the farming system^28^. The Kharif season (from June to September) corresponds to the rainy season fed by the South-West monsoon, when most of the agricultural area is cultivated, with rainfed crops including sorghum, maize, sunflower, marigold, as well as strictly irrigated crops, such as turmeric, onion, garlic, and banana. The Rabi season (from October to January) corresponds to the winter season fed by the North-East monsoon, when main crops are maize, horse gram, and vegetables. Finally, the Summer season (from February to May) corresponds to the hot and dry season, when only a fraction of the irrigable plots is cultivated^29^. This season is also the period during which reservoirs are usually empty and therefore desiltation is practicable. Red soils (Ferralsols and Chromic Luvisols), mainly characterized by coarse soil texture (sandy loam), cover the uplands and hillslopes, while black soils (Vertisols and Vertic intergrades), mainly characterized by finer soil texture (clay), are found mostly in the valley bottoms^30,31^. The Berambadi watershed has 5461 farming households in 12 villages^28^. The landholding is 1.2 ha on average, with 80% of small and marginal farms^28^. The unit of land holding is called “jeminu” in the local language, and consists of one or several adjacent plots cultivated by the same farmer^28^. A farmer can own several “jeminus”, in different locations, including in different villages. The Berambadi watershed contains 40 reservoirs with surface areas ranging from 0.1 to 49 hectares. Fourteen reservoirs have an area of less than 0.5 hectares, nine from 0.5 to 1 hectares, thirteen from 1 to 3 hectares and four above 3 hectares.

### Survey

We selected 66 farmers with experience in bottom sediment application to cropland. All were settled in villages belonging to the Berambadi watershed, but a few owned land slightly outside the watershed boundaries (Figure 1b). For logistical reasons, the survey was implemented in 3 phases, with the same translator: we first tested the questionnaire with 2 farmers in August 2021, then we interviewed 34 farmers from Mid-August to Mid-September 2021 and finally 30 farmers in February 2022.

The questionnaire (supplementary data 1) included 28 questions, plus one specific to farmers who had applied bottom sediment only one-time on a specific plot and five specifics to farmers who had applied bottom sediment several times on a specific plot. This enabled us to explore three main aspects of the practice of applying bottom sediments on farmland: 1) farmers’ reasoning (factors governing the decision, frequency, choice of reservoir...), 2) description of the practice (logistics, quantity spread and cost) and 3) expected benefits and induced adaptation of the cropping system (cropping choices, irrigation, organic and chemical fertilization).

We asked both closed-ended and open-ended questions to farmers. In the case of open-ended questions, farmers could provide one or more responses. The collected responses were then analysed, expressing the results as a percentage of the total number of answers (in this case the total is always equal to 100%) or of the total number of farmers surveyed (in this case the total may exceed 100%).

The sampling procedure depended on the availability of farmers and was restricted to farmers who already applied sediment on their fields. Farmers were interviewed on their own field or at home. Prior to each interview, farmers were informed about the aim and content of the research study and we formally asked verbally for permission to conduct the interview, take notes, translate and analyse the data anonymously. We assigned a unique and anonymous code to each of the farmers (Su1 to Su66). All interviewees gave informed consent for participation. No personal information is disclosed in this study. The research protocol wasn’t subject to formal review by an Institutional Review Board as it didn’t meet requirements for assessment (limited to health research).

The cost of bottom sediment application provided by the farmers corresponded to the prices on the year of application. We corrected these prices with the Indian annual inflation rate, using the consumer price index (CPI), defined by the Organisation for Economic Co-operation and Development (OECD) as the change in the prices of a basket of goods and services that are typically purchased by specific groups of households. These annual inflation rates were extracted from the database provided by the International Monetary Fund, World Bank and OECD Inflation CPI indicator (https://www.worlddata.info/asia/india/inflation-rates.php*)* and a CPI of 100 for 2021 was used for the correction. Prices were expressed both in Indian Rupees (INR) and US Dollar (USD) using an average exchange rate of 1 USD for 74 INR in 2021.

Finally, farmers used either tippers or tractors for bottom sediment transportation, both described in Supplementary Figure S1. As they expressed the quantity of bottom sediments they applied in terms of number of loads, we estimated the applied volume based on the dimensions of vehicles (Supplementary Data 2) and the number of loads.

### Sampling and physico-chemical analysis

**Fig. 2:**
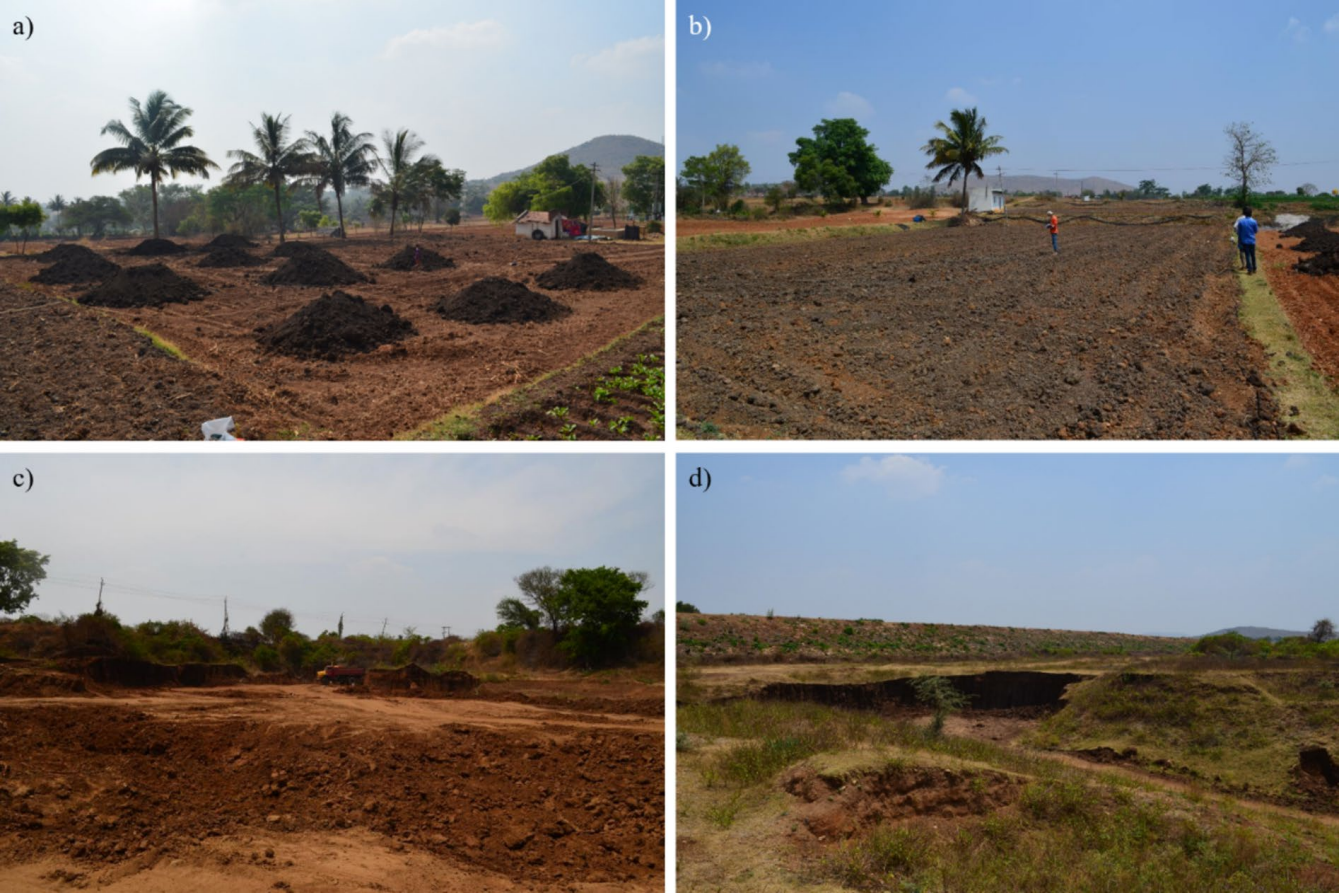
Illustration of (a) a plot where bottom sediment extracted from the Gundlupete Dam is deposited in pile to the field before mixing with the soil, (b) a plot where bottom sediment extracted from the Gundlupete Dam was recently mixed with the soil, (c) Gopalpura and (d) Kallipura reservoirs where sediment samples are extracted. Credit photo C. Gomez.

We collected twenty-six samples of soil and sediment during the dry season, in February 2021 (Fig. 1b), including four soil samples collected over plots recently mixed with bottom sediments (Fig. 2b), six sediment samples collected at the bottom of six empty reservoirs (Fig. 2c and d) and eight couples of [Soil & Sediment] in farm plots where sediment application was ongoing: the soil sample corresponded to the soil before sediment application, and the sediment sample was collected on piles deposited on the plot before mixing with the soil (Fig. 2a).

Each sample weighed about 1500 g. Each soil sample (*n* = 12) was composed of soil collected to a depth of 10 cm at five locations within a 10 × 10 m square at the centre of a cultivated plot, where the soil was plowed and homogenized. Each sediment pile sample (*n* = 8) was composed of sediment from at least 5 different piles of the same plot. Each bottom sediment sample (*n* = 6) was collected from an area of 2 × 2 m within each reservoir, from 0 to 1 m of depth. Finally, the 14 sediment samples (8 collected on piles and 6 on reservoirs) corresponded to 6 different reservoirs (Table 1).

**Table 1:** Number of sediment samples per reservoir.

Reservoir names	Number of samples collected on sediment piles	Number of samples collected in the reservoir
Gundlupete Dam	2	0
Kallipura	0	2
Gopalpura	2	1
Kunagahalli	1	1
Koothonur	2	2
Unknown	1	0

The samples were homogenized, air-dried and sieved at 2 mm prior to analysis. The classical physico-chemical soil properties of the 26 samples were determined following procedures^32^ described hereafter:

- Particle size distribution (clay -granulometric fraction < 2 μm, silt -granulometric fraction between 2 and 50 μm, sand -granulometric fraction between 0.05 and 2 mm) was determined according to the normalized protocol NF X 31–107 (by sieving and the Robinson pipette method);
- soil pH was determined according to the ISO standard 10,390;
- Cation Exchange Capacity (CEC) was determined using the cobaltihexamine chloride method according to the ISO standard 23,470 and NFX 31–130 and then the exchangeable K was determined by ICP-AEC analysis;
- Plant-available Phosphorus (P) was determined based on the Olsen method according to the normalized protocol NF ISO 11,263;
- Total Nitrogen (TN) was determined according to the ISO standard 13,878;
- Total Calcium carbonate (CaCO_3_) was determined according to the ISO standard 10,693;
- Total Carbon (TC) and Total Organic Carbon (TOC) were both determined according to the ISO standard 14,235;
- Organic Matter (OM) content and carbon to nitrogen ratio (C/N) were then calculated.

Statistical comparison of these physico-chemical properties of soil, sediment and soil recently mixed with sediments were conducted with R software^33^. The Tukey test was used to compare each group to all the others (Group 1: 8 soil samples; Group 2: 14 sediment samples; Group 3: 4 samples of soil recently mixed with bottom sediments) in a series of two-variable hypothesis tests^34^. The null hypothesis in each comparison is that both group means are equal; rejection of the null means that there is a significant difference between them. The differences were considered statistically significant when the p-value of the Tukey test was less than 0.05. As the Tukey test is based on an ANOVA, the assumption of homogeneity of variance and the assumption of normality of distributions have been checked first, and the Tukey test was performed only on properties respecting both conditions.

The non-parametric paired samples Wilcoxon test was used to analyse the soil property differences between soil and sediment considering the 8 couples of soil and sediment^35^. The differences were considered significant when the p-value of the Wilcoxon test was less than 0.05. To be applied, the samples have to respect the normality of distributions which was tested with the Shapiro-Wilk normality test^36^.

## Results

### Characterization of surveyed farmers and farms

The age of the 66 interviewed farmers ranged from 20 to 75 years, with an average age of 45 years. 79% of them own cows and apply their manure in their fields. All farms except one have access to groundwater irrigation. The size of the land owned ranged from 0.2 to 6.6 hectares, with a mean of 2.1 hectares, and small and marginal farms (< 2 ha) represented 59% of the total. This was relatively larger than the average landholding in the watershed^28^. Amongst the interviewed farmers, 31.8% own only one jeminu, while a majority (48.5%) own two, 12.1% own three and 7.6% more than three. This is comparable with the proportion found by Robert et al.^28^ in the whole watershed for irrigable farms (farmers owning only one jeminu represent 32% of irrigable farms and 63% of non-irrigable farms). We can assume that farmers interviewed in the present study belong either to the types “Mid-size irrigated farm owners” or “irrigating smallholders” of the typology proposed by Fischer et al.^37^ for this region.

The area of the 66 plots for which we documented sediment application ranged from 0.1 hectare to 2 hectares, with an average of 0.49 hectares. Only one of the studied plots had no irrigation facility. 85% of the studied plots were located within the Berambadi watershed boundaries (Fig. 1) and were well distributed over the watershed. Most of them belonged to the villages of Beemanbeedu (20%), Mallayanapurra (15%), Berambadi (11%) or Kallipura (9%).

### Farmers’ knowledge and practice of sediment application

We asked farmers how they learned about the practice of bottom sediment application. Although the question was open-ended, all farmers gave only one answer: 66% of them answered they learned from their neighbours, 17% by themselves, 11% from family members and 6% from other farmers including some from the neighbouring state (Tamil Nadu). Surprisingly, family was far from being the main channel for knowledge transmission in our study site, although bottom sediment application is a traditional practice in South India^20,22^.

**Fig. 3:**
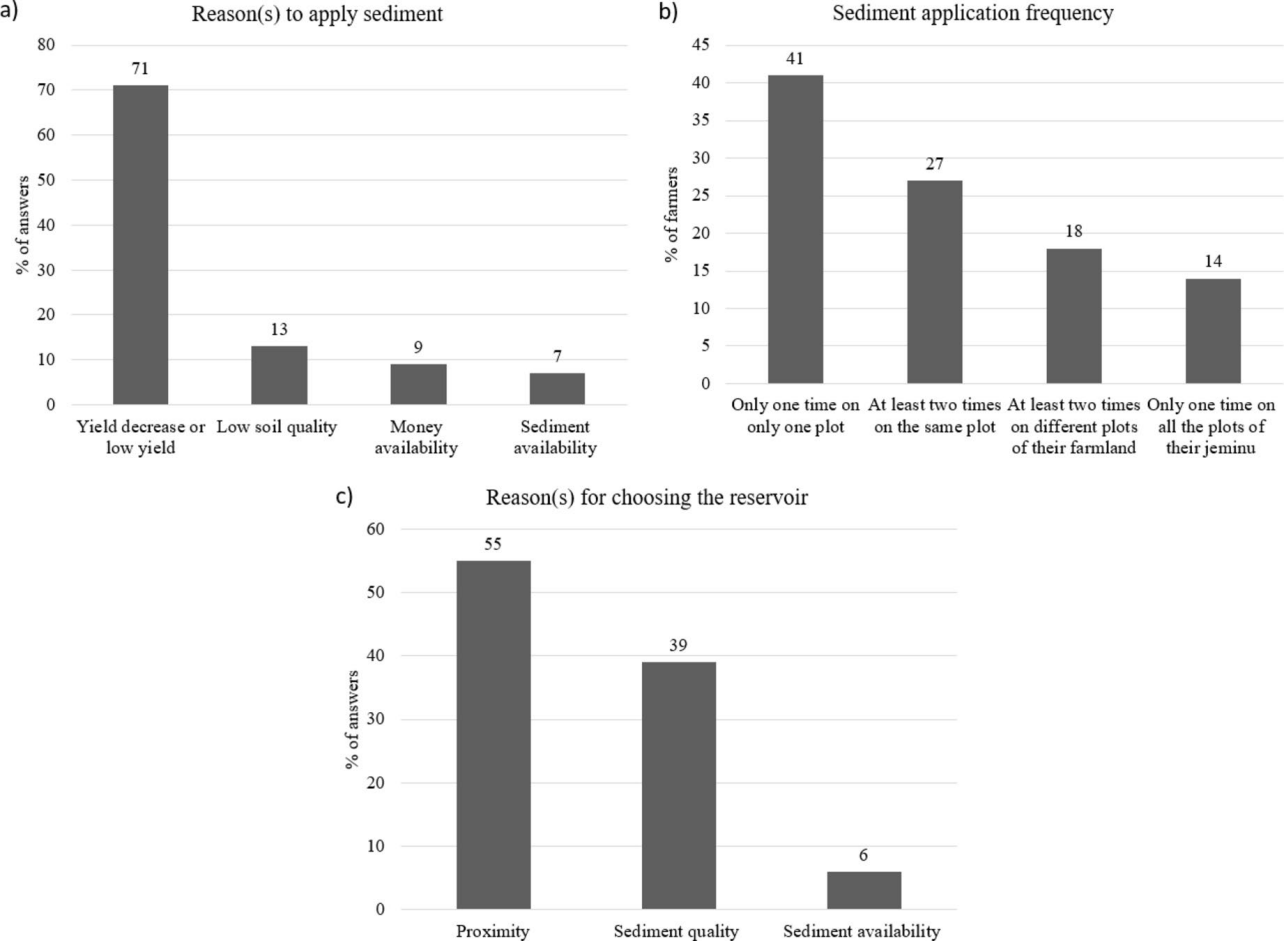
Description of the practice: (a) reason(s) to apply sediment (in % of answers), (b) frequency of sediment application (in % of farmers), (c) reason(s) for choosing the reservoir (in % of answers).

We asked farmers which reasons were triggering their decision to apply sediment on a specific year. The question was open-ended, and some farmers gave more than one reason. 71% of the answers were related to a yield decrease or low yield, 13% were related to a low soil quality, 9% were related to money availability and 7% to sediment availability (empty reservoir) (Fig. 3a). Clearly, the ability to access the resource (in terms of funding or sediment availability) was not the main driver of the decision, which was rather the need to restore the land’s productivity. 10 farmers mentioned spontaneously that it was necessary to apply sediment a few years after starting irrigation on a plot (estimated from 1 to 10 years depending on the farmers) as, to their point of view, repeated irrigations decrease both soil quality and crop yield. Indeed, irrigation allows increasing cropping intensity, with up to three crop cycles per year, compared to one or two in rainfed conditions^37^. This could lead to a decline of soil quality linked with biological degradation, nutrient depletion, soil compaction and physical degradation^38^, and therefore a decrease of yield.

We asked farmers about the dates and frequency of sediment application. The years of applications of bottom sediment ranged from 2001 to 2022, with a peak in 2017, which can be explained as the particularly weak monsoon of 2016 left all reservoirs empty, including the Berambadi reservoir, the largest one^39^. 41% of the farmers have applied bottom sediment only one time, on only one of their plots; 27% did it at least two times on the same plot, 18% did it at least two times but on different plots of their farmland and 14% applied bottom sediment only one time on all the plots of their jeminu (Fig. 3b). This may suggest that sediment application is essentially a one-off practice, and for the farmers who have repeated it at least twice the practice on the same plot, we found with very different time intervals between applications. While Osman et al.^16^ reported that “farmers believe that once applied, the impact on crop yield will remain for three years”, we were unable to highlight such a pattern from our survey.

We asked farmers their reasons for choosing the reservoir they extracted sediment from (open-ended question). 55% of the answers were related to the proximity, 39% to the sediment quality and 6% to the availability of sediment (empty reservoir) (Fig. 3c). It might suggest that logistics (including cost of transport) takes precedence over sediment composition in farmers’ decisions. Over the 66 plots, bottom sediments were extracted from 14 different reservoirs: most sediments were from Koothonur (44%), Kallipura (15%) and Deverakarre reservoirs (10.6%). The distance as the crow flies between the reservoirs and the fields where bottom sediments were applied ranged from 0.2 to 3 km with an average of 1.3 km. 45% of fields were located less than 1 km from the supply reservoir, 30% between 1 and 2 km away, and 25% between 2 and 3 km. The average distance in our study area was lower than the one observed in the state of Maharashtra by Zade et al.^40^, which was 2.4 km. Hence, while motorisation probably allowed a spatial expansion of the practice, we observed that a majority of farmers still selected the closest reservoir to collect sediment.

Finally, among the farmers who applied bottom sediment at least twice (either on the same field or on different fields of their farmland), 90% of them always used bottom sediment from the same reservoir. Of the three farmers who decided to use a different reservoir, only one cited sediment quality as a reason, as his crop yield had been lower than expected after the first application. The other two were forced to change as sediment extraction was not possible in the first reservoir, either because it was full of water or because all the sediment had already been extracted.

### Logistics and cost of sediment application

**Fig. 4:**
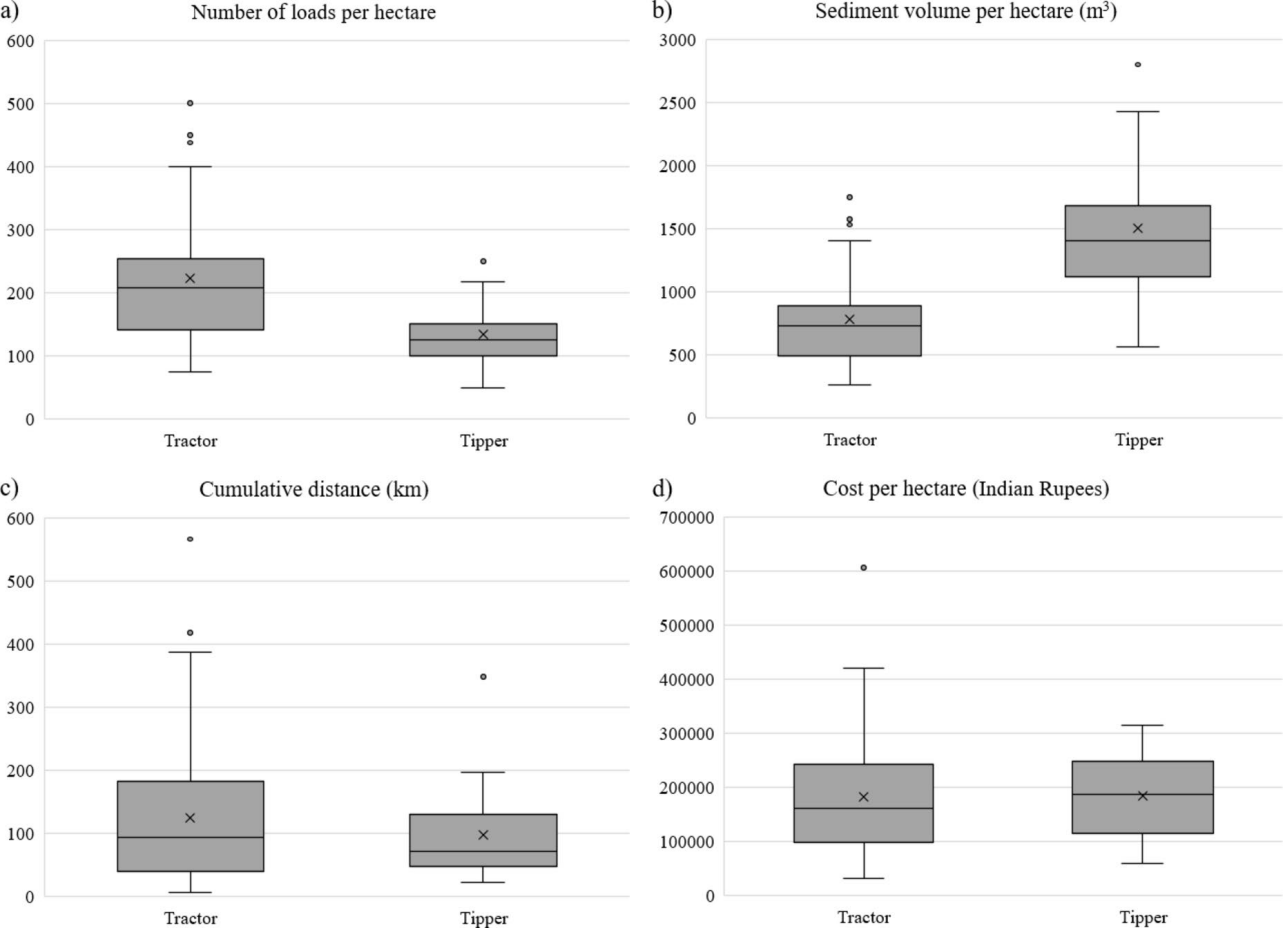
Boxplots of the (a) number of loads per hectare, (b) sediment volume (in m^3^) per hectare, (c) cumulative distance (in km) and (d) cost per hectare in Indian Rupees (average exchange rate between US Dollar (USD) and Indian Rupee (INR) was 1 USD for 74 INR in 2021) using either tractors or tippers.

For bottom sediment transportation, most farmers (73%) used tractors (Figures S1c and S1d) while the others (27%) used tippers (Figures S1a and S1b). This is probably because tractors are available in the villages, while tippers belong to private contractors involved in roadworks and are less easily accessible. The average size of the plot receiving sediments was similar for tractors (0.49 ± 0.29 ha) and tippers (0.51 ± 0.39 ha). The number of loads per plot was highly variable, ranging from 9 to 400 with an average of 114 (Coefficient of Variation = 79%) for tractors and from 20 to 300, with an average of 71 (CV = 90%) for tippers. While this variability was significantly reduced by normalizing the number of loads by the field area, it remained high, with a number of loads per hectare for tractors ranging from 75 to 500, with an average of 223 loads/ha (CV = 45%), and for tippers ranging from 50 to 250, with an average of 134 loads/ha (CV = 36%) (Fig. 4a). Given these load numbers and the estimated load capacities of the tractors and tippers (around 3.5 and 11.2 m^3^ respectively; Supplementary Data 2), the average sediment volume applied per hectare using tractors (780 m^3^/ha) was around half that applied with tippers (1500 m^3^/ha) (Fig. 4b). This result, together with the high variability observed between plots, suggests that there is no consensus among farmers in the region on the optimum dose of sediment to apply.

These sediment volumes per hectare correspond to an average thickness of 7.8 ± 3.5 cm and 15 ± 5.5 cm for tractors and tippers respectively. Zade et al.^40^ reported a similar range of magnitude in Maharashtra, where transport is mainly by tractors, with a sediment thickness of around 3 to 6 inches (7.6 to 15.2 cm).

Farmers used tippers for longer distances, with the median distance between the plot and the reservoir being 1.98 km and 0.98 km for tippers and tractors respectively. However, the cumulative distance (Fig. 4c), calculated as the product of the number of loads by the distance reservoir-field (one-way) was only slightly larger and more variable for tractors (from 7 to 565 km, with a mean of 124 km, CV = 95%) than for tippers (from 21 to 347 km, with a mean of 97 km, CV = 77%). This large variability suggests that the cumulated distance was not controlling the application rate.

The total expenditure for bottom sediment application includes only the rental of the machinery used for extraction from the reservoir, transport to the field and spreading. The sediment itself is free and no authorization is required for extraction. Most farmers paid the contractors out of their own savings (72%), while others took out a loan from other farmers (18%) or from a bank or a private company (6%). One farmer used both his savings and credit from other farmers, and one farmer did not answer the question. Finally, only one farmer benefited from a “Government” support to pay for the mechanical digger for extracting sediments from the reservoir. To explain the little use of bank loans, some farmers explained that banks would not grant loans for bottom sediment application. One farmer even admitted that a bank had given him a loan for crop cultivation, but that he had used the money for the application of bottom sediment.

The cost per plot of bottom sediment application indicated by farmers was highly variable, but on average very substantial (mean of about 90 000 INR per plot, i.e. around 1200 USD, CV = 95%). The mean cost per hectare was similar for both modes of transportation (182 000 INR/ha, i.e. around 2 460 USD, CV = 61%) compared to tippers (185 000 INR/ha, i.e. around 2 500 USD, CV 42%) (Fig. 4d). Considering the mean application rates (Fig. 4b), this means that applying a cubic metre of sediment is around twice more expensive with tractors than with tippers. It suggests that when farmers have the opportunity to access tippers, they prefer to apply more sediment than to reduce their expenses.

Farmers agreed with vehicle contractors mostly on a predetermined number of days of vehicle use (61%) or on the total cost (9%) – both equivalent to a total cost -while only 30% agreed on a predetermined number of round trips, which is equivalent to a predetermined application dose. This could explain why the cost per hectare was poorly to very poorly correlated with the number of loads (R of 0.54 and 0.28 for tractors and tippers, respectively) and the cumulative transport distance (R of 0.12 and 0.19 for tractors and tippers, respectively).

We are aware that all this quantitative information on the logistics of the practice, which calls on farmers’ memories, must be taken with caution. Surprisingly, however, farmers generally remembered the precise number of loads and the cost without hesitation, even when bottom sediment application had been carried out several years prior to the survey, whereas they had a much harder time remembering which crop was grown at the time. This could be explained by the fact that, as we pointed out above, sediment application is not a routine practice for them and, given its high cost, farmers remember its details well.

In summary, the wide variability in sediment application quantities and costs suggests that for most farmers, the objective is to apply as much sediment as possible with their available savings, in particular by choosing the closest reservoir, and not to reach a specific dose per hectare. When asked if they would have applied more sediment had it been possible, only 23% of the farmers answered that they would have put more had they have more money – and these were not the one who had put the lowest doses - while 77% answered that it was enough, confirming the lack of consensus on the objective dose.

### Expected benefit of the practice and induced adaptation of the cropping system

When asked how they thought bottom sediment application affected soil quality, farmers’ most spontaneous answer was that sediment application improved crop yield (43%) – which was not providing any specific mechanism. In this case, or when no spontaneous answer was given, we suggested several options (supplementary data 1). Answers included increased soil water storage (28%), a similar benefit to manure (13%), improved soil texture and nutrient content (13%) and reduced risk of crop disease (2.6%). Aggregating these answers, we found that two third (68%) of the farmers suggested, in at least one of their answers, an impact on soil physics (soil water storage, texture and/or action similar to manure), while one third (33%) suggested an impact on soil chemistry (soil nutrients content and/or action similar to manure) and about a quarter (27%) did not provide any specific mechanism (Table 2). 77% of the farmers who mentioned an impact on soil chemistry also mentioned an impact on soil physics.

None of the farmers interviewed reported having introduced new crops after sediment application, except where this was concomitant with the installation of an irrigation system on the plot. Most of them were therefore able to compare their cropping practices for the same crops before and after sediment application (Table 2). The use of manure was unchanged for most farmers (64%), probably because almost all farmers use the manure produced by their own cows. A majority of farmers declared that they decreased their use of chemical fertilizer (58%) and the frequency of irrigation (53%). A reduction of chemical fertilizer use following application of bottom sediment was reported by several authors^16^, although Zade et al.^40^ reported that many farmers “were reluctant to reduce their input as they felt they had invested significant money in silt application and feared loss of their investment in case of failed crops*”.* Decrease of irrigation frequency following sediment application is less documented in the literature.

Interestingly, farmers who did not identify a specific mechanism for the impact of sediment on soil were less likely to modify their cropping practices (Table 2). On the contrary, farmers who cited an impact on soil chemistry were significantly more likely to reduce their chemical fertilization, while farmers who cited an impact on soil physics were significantly more likely to reduce their irrigation (Table 2). This underlines the importance of helping farmers build their capacity to better understand the impact of the sediment application, so that they can better integrate it into their farming practices.

**Table 2:** Expected benefit of the practice and induced adaptation of the cropping system. “Chemistry” sums up answers related to soil nutrients content and action similar to manure, “physics” sums up answers related to soil water content, texture and action similar to manure. “N/A*”* refers to cases where either the question was not applicable (i.e. Farmers who never used manure nor chemical fertilizer, or who accessed irrigation from the year of their first sediment application) or when the change in practice was independent of the application of bottom sediment (i.e. Decrease of irrigation due to a Decrease of pump yield).

	Expected benefit (% farmers)	Adaptation of the cropping system following bottom sediment application (% farmers)											
		Chemical fertilizer				Manure				irrigation			
		less	same	more	*N*/A	less	same	more	*N*/A	less	same	more	*N*/A
**All farmers**		*58*	*39*	*0*	*3*	*15*	*64*	*3*	*18*	*53*	*41*	*0*	*6*
**unspecified**	27	*50*	*37.5*	*0*	*12.5*	*19*	*62.5*	*6*	*12.5*	*19*	*62*	*0*	*19*
**Chemistry**	33	*68*	*32*	*0*	*0*	*14*	*63*	*0*	*23*	*59*	*36*	*0*	*5*
**Physics**	68	*55*	*44*	*0*	*0*	*9*	*67*	*2*	*22*	*69*	*29*	*0*	*2*

### Physico-chemical characteristics of sediment and soil

**Fig. 5:**
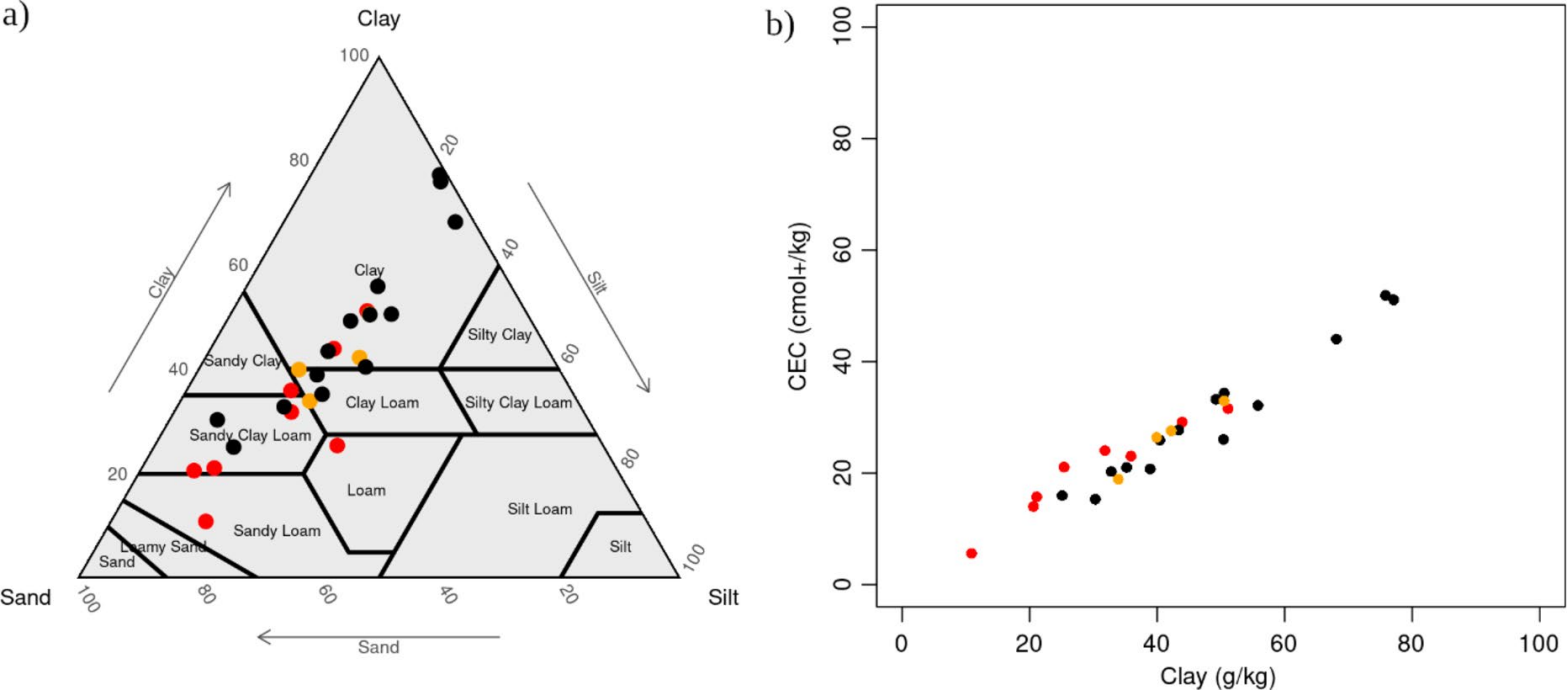
(a) Distribution of the 26 soil and sediment samples across the USDA soil textural triangle and (b) relation between CEC and clay for the 26 soil and sediment samples (red dots: soil samples collected over plots before bottom sediment application; black dots: bottom sediment samples; orange dots: soil samples collected over plots recently mixed with bottom sediments).

The texture of the 14 sediment samples varied, according to the USDA soil textural triangle, from Sandy Clay Loam (SaClLo) to Clay (Cl) (black dots on Fig. 5a; Supplementary Table 1). While their silt contents were around 20%, the clay and sand contents were very variable. This result is in accordance with those of Braga et al.^13^ who highlighted a significant variability of particle sizes across four reservoirs in Brazil. To the contrary, surveys carried out in India seems to display a smaller variability across reservoirs within the same region, but textures varied broadly across regions: in Andhra Pradesh, sediments from 12 reservoirs belonged all to the Clay class^16^; in Maharashtra, sediments from 11 reservoirs belonged to the Silt Loam and Silty Clay Loam classes^15^; in Rajasthan, sediments from 10 reservoirs belonged to the Sandy Clay Loam, Clay Loam and Sandy Clay classes^41^.

Soil samples collected in the plots before bottom sediment application were also characterised by a large range of textures, from Sandy (Sa) to Clay (Cl) (red dots on Fig. 5a; Supplementary Table 1). Interestingly, although the soil samples were on average more sandy and sediment samples more clayed, their range of variation were largely overlapping (Fig. 5a). Finally, soils recently mixed with bottom sediment were characterised by a narrower range of textures, from Sandy Clay Loam (SaClLo) to Clay (Cl) (orange dots, Fig. 5a; Supplementary Table 1). For the three groups of samples (soil, sediment and soil mixed with sediment) we found a strong correlation between CEC and clay (R of 0.96, 0.97 and 0.97 for soil, sediment and soil mixed with sediment, respectively; Fig. 5b).

Chemical properties of sediments were within the range reported by Osman et al.^16^ in peninsular India. They were more variable than those of soils and mixtures, except for P and K content (Supplementary Figure S2). We could perform the Tukey test only on clay, silt, sand, CEC, TOC and OM, as the other parameters did not fulfil the necessary assumptions for processing an ANOVA (homogeneity of variances and normality of distributions). A significant difference between soil and sediment (*p* < 0.05) was observed for clay and sand (Supplementary Figure S2). Sediments had a higher clay content and a lower sand content than soils (soils mixed with sediments being intermediate, Supplementary Figure S2), as also observed by Bhanavase et al.^42^ and Canet et al.^14^. As a consequence, the increase of clay content due to sediment application may induce an increase of available water content (AWC) of the plowed horizon, as observed by Bhanavase et al.^42^ and Osman et al.^16^.

**Fig. 6:**
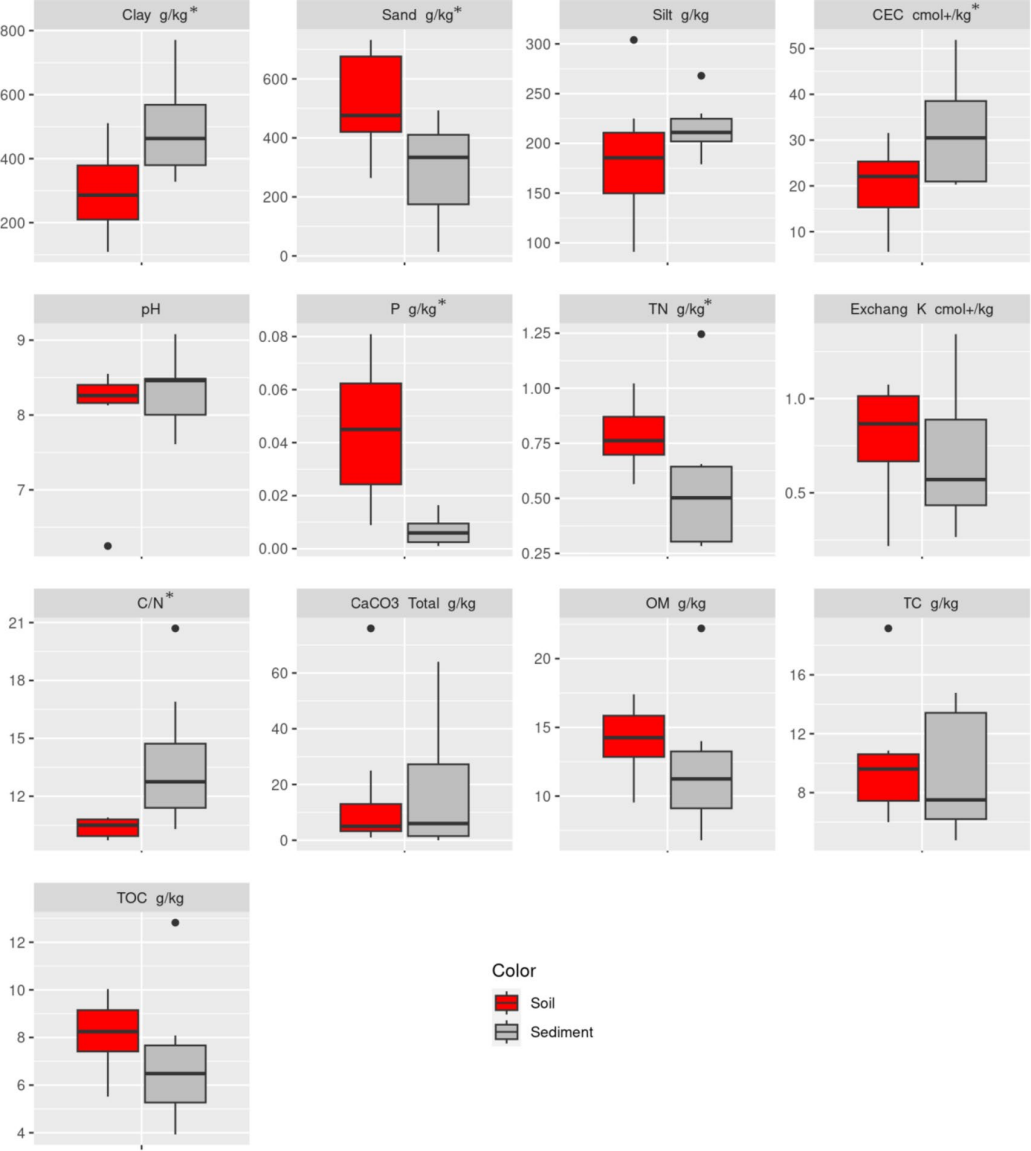
Distribution of the physico-chemical properties of the 8 couples of [Soil & Sediment]. * indicates a p-value < 0.05 (calculated with the Wilcoxon test).

To go further, we analysed the soil property differences between soil and sediment for the 8 couples of soil and sediment collected in farmer’s plots. The non-parametric paired samples Wilcoxon test was performed on all properties as they all respect the normality of distributions assumptions. A significant difference between soil and sediment (*p* < 0.05) was observed for clay and sand (as previously) and also for P, CEC, TN and C/N (Fig. 6). Available P and TN content were significantly lower in sediments than in soils (Fig. 6). A significant difference between soil and sediment was also observed for CEC (Fig. 6) with higher CEC in sediment than soil. Finally, we found a significant difference between soil and sediment for the C/N ratio (Fig. 6) with higher C/N in sediment than in soil.

## Discussion

### A very heterogeneous practice that only wealthy farmers can afford

Our survey clearly showed that the reason triggering the decision to apply bottom sediments in their plots was, for most farmers, a perceived drop in crop yield (Fig. 3a). In spite of this apparent consensus, the large quantity and the large variability of bottom sediments applied to fields (Fig. 4b) is one of the most striking result from our study. To illustrate this, if we consider a plowing depth of 30 cm (which is a maximum in the region), bottom sediment applied by tractor would constitute from 8.7 to 58% of the final plowing depth, while sediment applied by tipper would constitute from 18.6 to 93% of the final plowed horizon. Hence, in the Berambadi watershed, depending on the volume applied and the plowing depth, this practice can fall under the heading of soil amendment or may form anthropogenic soils^43^. However, this wide range of application rates seems to result not from specific pre-determined objectives, but rather from a combination of available budget and logistical factors such as distance from the reservoir or access to tippers.

Given the cost of the practice (around 90,000 INR per plot, i.e. around 1200 USD), it is definitely out of reach for farmers without access to irrigation. Even for irrigated farms, whose annual income varies between 60,000 and 700,000 INR (i.e. between around 810 and 9450 USD) per family worker^37^, and given that it is essentially financed by savings, we can assume that only the most affluent can afford it, as was also found in a recent study in Andhra Pradesh^22^. Consequently, it is likely that, under current conditions, this practice contributes to increasing inequalities between farmers, in addition to the inequalities typically caused by differential access to groundwater irrigation^37,44^.

### Little-shared local knowledge that determines the choice of whether or not to adapt farming systems

The lack of consensus among the farmers on the role of sediment on soil properties (Table 2), despite the scale of the investment involved (Fig. 4d), is one of the unexpected results of our survey. One possible explanation would be that the soil functions affected could vary according to the application rates and to the physico-chemical properties of soils and sediments. Another hypothesis is that the ancestral knowledge acquired over centuries of practice^20,23^ has not been passed on to the current generation. This could be either due to the wide time gap between the decline of the traditional bullock-based practice^20,22^ and the current motor-based one, or because this knowledge was not relevant to inform current practices, characterized by huge application rates that would have been unthinkable before motorization.

Importantly, farmers identifying a specific role of sediments were more likely to adapt their practices accordingly, whether by reducing fertilizer application or irrigation frequency, or both (Table 2). This underlines the importance of fostering the exchange of knowledge between farmers and, for researchers, of evaluating it scientifically. Interestingly, farmers did not accompany this practice with changes in crop choice. This suggests that, unlike other input-saving techniques, such as micro-irrigation which often result in higher resource consumption due to changes in farming systems^45,46^, bottom sediment application has real potential to reduce the dependence of farming systems on external inputs.

### Impact on soil properties: an amendment and not a fertilizer

Despite the large variability of their physico-chemical properties, our study confirms that bottom sediment has the potential to increase the soil clay content within the plowed horizon (Fig. 6; Supplementary Figure S2), as reported in several studies^14,42^. As noted by Canet et al.^14^, an increased water storage capacity in the plowed horizon, subsequent to sediment application, would reduce the need for irrigation, especially for shallow-rooted crops. This aligns with the perception of a majority of farmers who reduced their irrigation frequency after applying sediment (Table 2). A more extensive sampling campaign would be necessary to quantify the increase of clay content after mixing, to better document the variability in sediment properties within and across reservoirs, and identify the factors that govern it (position of the reservoir in the river network, proximity to forest.). Similarly, a quantification of the decrease of irrigation frequency declared by farmers, and of the improvement of the water use efficiency would require specific monitoring.

More surprisingly, we also found that sediments were not likely to improve the organic carbon nor the macronutrient (C, N, P) content of the soil. In fact, they are likely to worsen it, especially for N and P (Fig. 6; Supplementary Figure S2). This seems to contradict not only the several studies which consider sediments as a source of macronutrients^13,14,16,42,47,48^ but also farmers’ beliefs, as more than half of them declare reducing the fertilizer use following sediment application. There are two possible explanations for this apparent contradiction. The first is that sediment application, by increasing soil clay content, would improve CEC (Figs. 5b and 6) and water-holding capacity, thereby improving fertilizer use efficiency. The other is linked to the well-documented over-fertilization of groundwater-irrigated agricultural systems in India, in particular the huge excess of nitrogen^49^ leading to massive nitrate pollution of groundwater^50,51^. Possibly, farmers’ belief that sediment application improves soil nutrient content is rooted in the ancestral experience of the practice, when it was always accompanied by manure input, and it is reinforced by their observation of the absence of nutrient deficiency following application. If this is true, it could serve a basis to raise awareness among farmers of their over-fertilization practices. Finally, another hypothesis that remains to be tested is that sediment application improves the micronutrient content of the soil, and therefore the crop nutrient status. In any case this finding has to be confirmed with more soil and sediment analysis from different reservoirs in this region and beyond.

### The way forward

This study disproves our hypothesis that the local knowledge of farmers spontaneously resorting to this ancestral practice could serve as a basis for the development of a science-based rationalization. At the same time, it confirms that bottom sediment reuse on agricultural land is a promising agroecological practice, in the sense of Wezel et al.^52^, as it can increase the circularity and sustainability of cropping systems through improved irrigation and fertilization efficiency. However, to fully exploit its potential, we would need to considerably improve our knowledge in three main directions and over different contexts.

Firstly, it is essential to build a scientific rationale to help farmers define an optimal application rate. This involves better characterization of sediment properties and their heterogeneity as well as the processes that govern it. This would help explain the low macronutrient content of the sediments, which could be due, for example, to their origin in the watershed (deep soil by gullying rather than from topsoil by sheet erosion) or to in-lake processes. It also involves a deeper understanding of the impacts of these different sediment types on soil functions (physical, chemical and biological) and their persistence in time. This knowledge needs to be translated into simple indicators, which could enable farmers to decide on their dose according to the properties of the sediment and the soil that receives it. Optimum application rates would not only enable farmers not to overspend on a costly practice, but also to balance positive (on soil physics) and potentially negative (on soil nutrient content) impacts.

Secondly, the potential adverse effects of reusing sediments in agriculture need to be carefully assessed. These could be related to the recycling and accumulation in soils of pollutants, such as heavy metals, toxic chemicals or microplastics^12,53^, or to the risk of increasing salinization in groundwater-irrigated soils with high water holding capacity^54^. In addition, the impact of this practice on greenhouse gas emissions, whether due to the high motorization involved or to changes in soil functions, needs to be assessed.

Finally, the sustainability of this practice needs to be assessed on a territorial scale. Given the massive extraction of sediments made possible by the availability of heavy machinery, it is possible that extraction is currently much higher than the rate of sediment deposition in the reservoirs. We therefore need to define the volume available in reservoirs and a sustainable extraction rate. More importantly, in the current situation, it is likely that bottom sediment reuse is in fact a transfer of fertility from poorer farmers, who cannot invest in erosion control structures, to the richer ones. Ideally, bottom sediment reuse should be managed collectively, to ensure sustainability and equity. Unlike groundwater resources, whose “invisibility” often prevents collective management^55,56^, bottom sediments could be more easily managed as a common resource.

## Conclusion

This study, based on both a survey of farmers and the physico-chemical analysis of soil and bottom sediment samples in a watershed in Karnataka, South India, disprove our hypothesis that the local knowledge of farmers spontaneously resorting to this ancestral practice could serve as a basis for the development of a science-based rationalization. Indeed, we found a wide diversity of application rates, ranging from light soil amendment to the creation of anthropogenic soils, and no consensus on the specific mechanism of sediment impact on the soil. Nevertheless, our results provided a better understanding of the agricultural practice of reusing reservoir bottom sediments on cropland as we found that this practice is likely to significantly improve soil physical properties such as water holding capacity and cation exchange capacity, which might improve irrigation and fertilization efficiency. Finally, given the great agroecological potential of this practice in terms of resource circularity and sustainability of cropping systems, we advocate further studies to (i) understand mechanisms of sediment impact on soil functions which will allow to define optimal application rates, (ii) assess potential risks and (iii) promote collective management of the resource.

## Electronic Supplementary Material

Below is the link to the electronic supplementary material.

Supplementary Material 1

Supplementary Material 2

Supplementary Material 3

## Data Availability

Data sets generated during the current study are available from the corresponding author on reasonable request.
