# Supplementary figures and images for: Reuse of bottom sediment from reservoirs to cropland is a promising agroecological practice that must be rationalized
Source: Sci Rep. 2025 Mar 4;15:7523. doi: 10.1038/s41598-025-92206-2 (PMC11876658; doi:10.1038/s41598-025-92206-2)

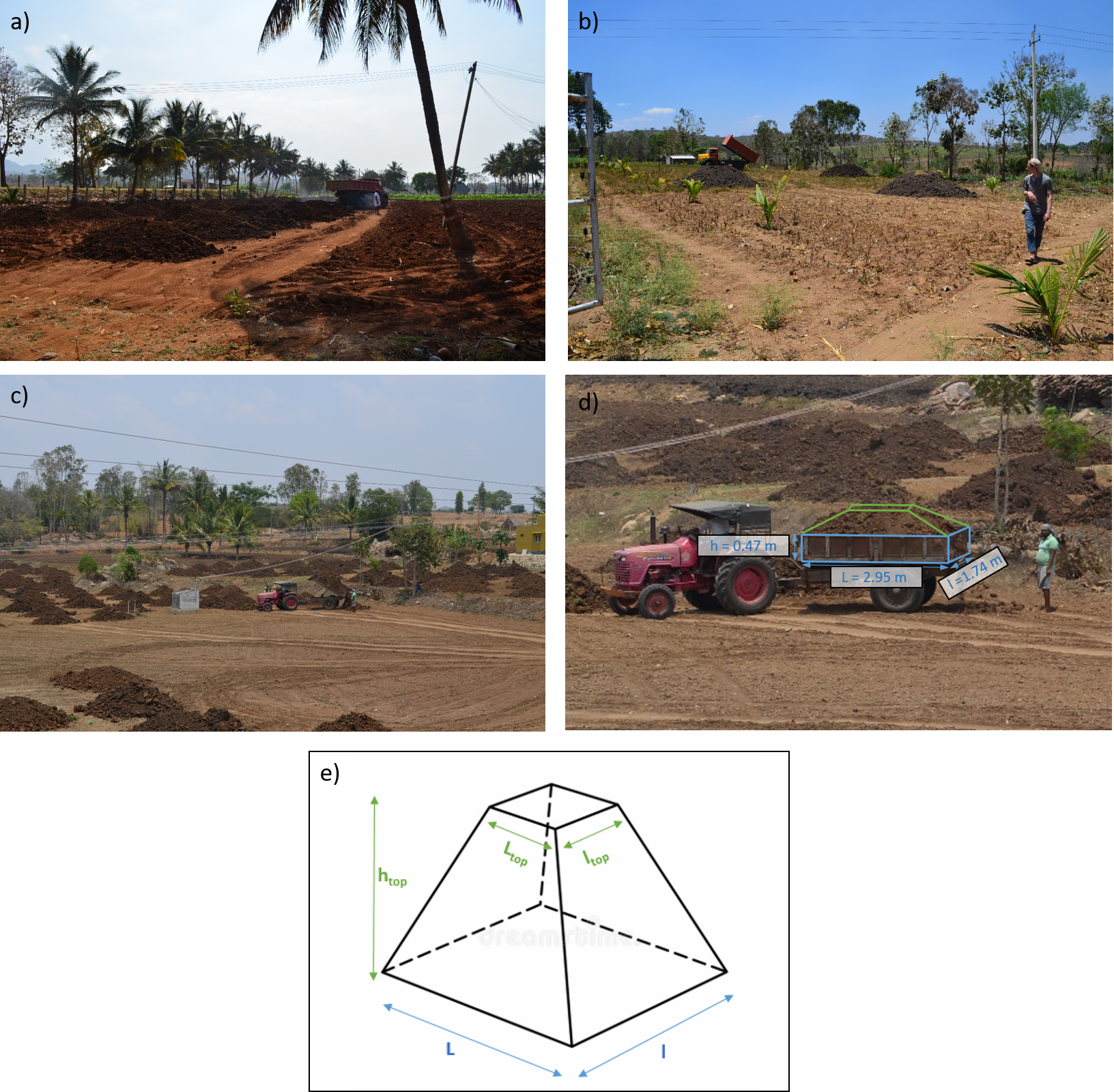

Supplement: Supplementary file 1 — Supplementary Material 1 [file 41598_2025_92206_MOESM1_ESM.png]

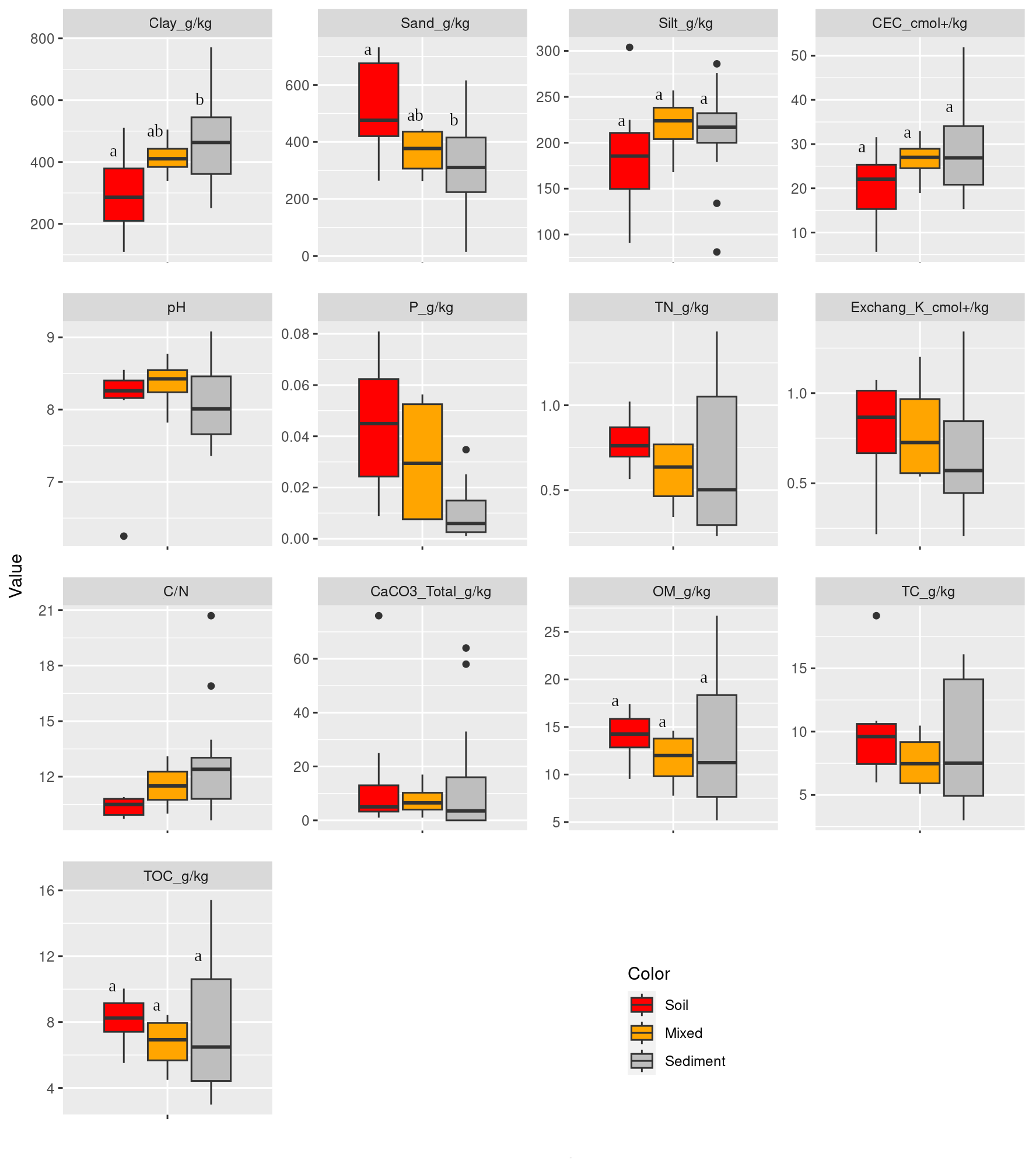

Supplement: Supplementary file 2 — Supplementary Material 2 [file 41598_2025_92206_MOESM2_ESM.png]
