# Supplementary material for: Reuse of bottom sediment from reservoirs to cropland is a promising agroecological practice that must be rationalized
Source: Sci Rep. 2025 Mar 4;15:7523. doi: 10.1038/s41598-025-92206-2 (PMC11876658; doi:10.1038/s41598-025-92206-2)

# **Supplementary materials for**

**Reuse of bottom sediment from reservoirs to cropland is a promising agroecological practice that must be rationalized**

Gomez^1 ,2^ C., Amelin^2,3^ J., Coulouma^1^ G., Gaab^1 ,2^ J., Dharumarajan^4^ S., Riotte^2,3^ J., Sekhar^5^ M., Ruiz^2,6,7,^* L.

^1^ LISAH, Univ Montpellier, IRD, INRAE, Institut Agro, AgroParisTech, Montpellier, France

^2^ Indo-French Cell for Water Sciences, ICWaR, Indian Institute of Science, Bangalore, India

^3^ Univ.Paul-Sabatier, GET, IRD, CNRS, Toulouse, France

^4^ ICAR‐National Bureau of Soil Survey and Land Use Planning, Hebbal, Bangalore, India

^5^ Indian Institute of Science, Civil Engineering Department, Bangalore, India

^6^ SAS, INRAE, Institut Agro, Rennes, France

^7^ G-EAU, INRAE, Univ Montpellier, AgroParisTech, Cirad, IRD, Institut Agro, Montpellier, France

* corresponding author : laurent.ruiz@inrae.fr

**Supplementary data 1: Survey conducted in Berambadi catchment, Karnataka, India in 2021 and 2022.**

Start the interview by describing the purpose of the survey: for academic research only, accurate answers are important and gratefully acknowledged, etc.

We are going to ask you some questions about your plot, your practices and mainly the tank sediment application.

All answers will be treated confidentially.

|  | **Sub-questions** | **Suggested answers** | **Free answer** |
| --- | --- | --- | --- |
| Survey Date |  |  |  |
| Survey Hour |  |  |  |
| Interviewer Name(s) |  |  |  |
| **I. General information** | | | |
| Name of household head |  |  |  |
| Age and education of the head of household |  |  |  |
| Village of household residence |  |  |  |
| Total owned land (acres) |  |  |  |
| DISHAANK number of the specific plot |  |  |  |
| Cadastral number and Village name of the specific plot |  |  |  |
| GPS coordinates of the specific plot |  |  |  |
| Topsoil color of the specific plot |  |  |  |
| Type of neighboring soil color |  |  |  |
| **II Sediment application practice over the plot** | | | |
| When was your first sediment application on the plot? |  |  |  |
| Did you use Tractors or Tippers? |  |  |  |
| Number of Tractors or Tippers |  |  |  |
| Volume of applied sediment |  |  |  |
| Estimation of plot area |  |  |  |
| Estimation of Jeminu area |  |  |  |
| Total Cost (to apply sediment) |  |  |  |
| How did you agree with the contractor? |  | *Pre-determined number of tractor/tipper; Pre-determined duration.* |  |
| Did you adjust (and pay) depending on the realization? |  | *Yes or No* |  |
|  | If “yes”, based on what? |  |  |
| How do they arrange payment? (loan, credit or ...) |  |  |  |
| Sediment origin (which tank) |  |  |  |

|  | **Sub-questions** | **Suggested answers** | **Free answer** | |
| --- | --- | --- | --- | --- |
| **III Knowledge about the practice** | | | | |
| How did you learn about this practice? |  | *Personal experience;*  *Familial tradition; Observations of neighbors.* | |  |
| How do tank sediments act on soil quality? |  | *Same as fertilizers; Same as manure;*  *Limit erosion (soil loss);*  *Improve soil water storage;* | |  |
| Do you think there is a difference of sediment quality, between tanks in the region? |  | *Yes or No* | |  |
|  | If “yes”, Which one(s) is(are) the best? |  | |  |
|  | If “yes”, Which one(s) is(are) the worst? |  | |  |
|  | What are these differences, and what function(s) of the tank sediment they affect? | *Replace fertilizers; Replace manure;*  *Limit erosion; Improve water storage;* | |  |

| **IV Experience** | | | |
| --- | --- | --- | --- |
| Why did you choose this tank? |  | *Distance to the tank; 󠄀*  *Sediment quality; Access authorization.* |  |
| Why did you choose to put tank sediment this specific year? |  | *Low yields;*  *Money availability;*  *Tank access authorization;*  *Empty tank;*  *Last season was too dry/too rainy;*  *Less rains but extreme/heavy.* |  |
| Do you plan to put again tank sediment on this plot in future? |  | *Yes or No* |  |
|  | If "yes", When or what will make you decide to do again? | *Fixed frequency; Low yields;*  *Money availability;*  *Tank access authorization; Empty tank; Depend on climate (rains, dryness).* |  |
|  | If "no", Why? | *Not efficient;*  *I have done enough;*  *Too expensive;*  *No Tank access authorization;*  *Full tank.* |  |

***Specific question for farmers applying sediment for first time***

| Why didn't you do it before? |  | *Yields ok; 󠄀*  *Not enough money;*  *No access to the tank;*  *Climate was ok.* |  |
| --- | --- | --- | --- |

***Specific questions for farmers having appliedat least 2 times***

| When did you start to put sediment on this plot? (year) |  |  |  |
| --- | --- | --- | --- |
| When were the other times? (dates or frequency) |  |  |  |
| How many times did you put sediment (including this year)? |  |  |  |
| Why did you choose this frequency? |  | *Low yields;*  *Money availability; Tank access authorization; Empty tank; Depend on climate (rains, dryness).* |  |
| Do the sediments always come from the same tank? |  | *Yes or No* |  |
|  | If "no", Name of the other tank(s) |  |  |
|  | If "no", Why several tanks? | *Depend on expected functions; Money availability; Tank access authorization; Empty tank.* |  |
|  | If "no", Do you see differences about soil quality between these tanks? |  |  |
|  | If "yes", Why do you always choose the same tank? | *Distance to the tank; 󠄀*  *Sediment quality; 󠄀 Access authorization. 󠄀* |  |

|  | **Sub-questions** | **Suggested answers** | **Free answer** |
| --- | --- | --- | --- |
| **V. Agricultural Practices on the plot** | | | |
| Do you practice irrigation on the plot? |  | *Yes or No* |  |
|  | If "yes", Since when? |  |  |
| Previous crop (before sediment application) |  |  |  |
| Next crop (so after sediment application) |  |  |  |
| Is it the first time you will do (are doing) this crop? |  | *Yes or No* |  |
| Will you change your practice regarding chemical fertilizer? |  | *plus / equal / less* |  |
| Will you change your practice regarding organic fertilizer? |  | *plus / equal / less* |  |
| Will you change your practice regarding irrigation? |  | *plus / equal / less* |  |

| **VI. On the Jeminu (if Plot < Jeminu)** | | | |
| --- | --- | --- | --- |
| Do you have the same practice (=sediment application) on all your Jeminu? |  | *Yes or No* |  |
|  | If "no", Why? | *Just try on one plot? 󠄀 Not enough money? 󠄀 Yield ok on others plots ?󠄀 󠄀* |  |

| **VII. Practices at household level** | | | |
| --- | --- | --- | --- |
| Do you have cows? |  | *Yes or No* |  |
|  | If "yes", How many cows do you have? |  |  |
|  | if "yes", How much manure per year ? |  |  |
|  | if "yes" Do you use your own manure over your plots? |  |  |
|  | if "yes", Do you sell manure? |  |  |
|  | If you sell manure, do you sell all or only a part? |  |  |
|  | If you sell manure, How much per year do you sell ? |  |  |
| Do you buy organic fertilizer (manure)? |  | *Yes or No* |  |
|  | If “yes”: How much per year do you buy fertilizer? |  |  |
| How many OTHER jeminus do you cultivate? |  |  |  |

if Number of Jeminus <= 3 (other than the studied plot)

|  | Jeminu 1 | Jeminu 2 | Jeminu 3 |
| --- | --- | --- | --- |
| Irrigated or Rainfeld? |  |  |  |
| Owned or Leased? |  |  |  |
| Surface (acres) |  |  |  |
| Sediment application ? |  |  |  |
| If sediment application: Sediment Origin? |  |  |  |
| If sediment application: date of first Sediment application |  |  |  |
| If sediment application: Frequency |  |  |  |
| Manure application (yes / no) |  |  |  |

if Number of Jeminus > 3 (other than the studied plot)

|  | Irrigated | | Rainfeld | |
| --- | --- | --- | --- | --- |
| Number of Jeminus |  | |  | |
| Surface (acres) |  | |  | |
| Sediment application ? | yes | no | yes | no |
| If sediment application: Sediment Origin? |  |  |  |  |
| If sediment application: Since  when? |  |  |  |  |
| If sediment application: Frequency |  |  |  |  |
| Manure application (yes / no) |  | |  | |
| Owned or  Leased |  | |  | |

**Supplementary data 2:**

The width (l), length (L) and height (h) of a tractor trailer are 1.74, 2.95 and 0.47 m, respectively (Supplementary Figure 1d). Considering these dimensions and a top part (highlighted by green lines on Supplementary Figure 1d) as a truncated pyramid with a width (l_top_), length (L_top_) and height (h_top_) estimated around 0.40, 0.80 and 0.47 m respectively (Supplementary Figure 1e), the carrying capacity of one tractor is estimated about 3.45 m^3^. The width (l), length (L) and height (h) of a tipper trailer are 2.52, 4.16 and 0.85 m, respectively. Considering these dimensions and a top part as a truncated pyramid with a width (l_top_), length (L_top_) and height (h_top_) estimated around of 0.7, 1 and 0.5 m respectively, the carrying capacity of one tipper is about 11.22 m^3^. So a tipper load represents 3.25 times a tractor load.

**Supplementary Table 1:** Summary statistics of soil properties of the 26 samples, grouped by types: soil before sediment application, soil recently mixed with sediment and sediment collected over tanks or piles.

|  | **Soil before sediment application** | | | | **Soil recently mixed with sediment** | | | | **Sediment (from tank or piles)** | | | |
| --- | --- | --- | --- | --- | --- | --- | --- | --- | --- | --- | --- | --- |
|  | **min** | **max** | **mean** | **median** | **min** | **max** | **mean** | **median** | **min** | **max** | **mean** | **median** |
| **Clay (g/kg)** | 109.00 | 511.00 | 300.88 | 286.00 | 339.00 | 505.00 | 416.25 | 410.50 | 251.00 | 771.00 | 480.71 | 463.00 |
| **Silt (g/kg)** | 91.00 | 304.00 | 184.75 | 185.50 | 168.00 | 257.00 | 218.25 | 224.00 | 81.00 | 286.00 | 210.93 | 217.00 |
| **Sand (g/kg)** | 264.00 | 732.00 | 514.38 | 476.00 | 263.00 | 445.00 | 365.50 | 377.00 | 14.00 | 616.00 | 308.36 | 310.50 |
| **CEC (cmol+/kg)** | 5.61 | 31.56 | 20.53 | 22.06 | 18.91 | 32.97 | 26.47 | 27.00 | 15.32 | 51.88 | 29.99 | 26.88 |
| **pH** | 6.25 | 8.55 | 8.06 | 8.26 | 7.82 | 8.77 | 8.36 | 8.43 | 7.36 | 9.08 | 8.06 | 8.01 |
| **TN (g/kg)** | 0.56 | 1.02 | 0.79 | 0.76 | 0.34 | 0.78 | 0.60 | 0.64 | 0.23 | 1.43 | 0.65 | 0.50 |
| **P (g/kg)** | 0.01 | 0.08 | 0.04 | 0.04 | 0.01 | 0.06 | 0.03 | 0.03 | 0.00 | 0.03 | 0.01 | 0.01 |
| **K (cmol+/kg)** | 0.22 | 1.07 | 0.79 | 0.87 | 0.54 | 1.20 | 0.80 | 0.73 | 0.21 | 1.34 | 0.68 | 0.57 |
| **Total CaCO3 (g/kg)** | 1.00 | 76.00 | 17.86 | 5.00 | 1.00 | 17.00 | 7.75 | 6.50 | 2.00 | 64.00 | 22.33 | 13.00 |
| **TC (g/kg)** | 6.00 | 19.15 | 10.02 | 9.60 | 5.09 | 10.48 | 7.62 | 7.47 | 2.99 | 16.10 | 9.23 | 7.51 |
| **TOC (g/kg)** | 5.52 | 10.03 | 8.15 | 8.25 | 4.49 | 8.44 | 6.69 | 6.93 | 2.99 | 15.42 | 7.51 | 6.48 |
| **C/N** | 9.72 | 10.90 | 10.37 | 10.50 | 10.00 | 13.10 | 11.53 | 11.50 | 9.64 | 20.70 | 12.71 | 12.40 |
| **OM (g/kg)** | 9.54 | 17.40 | 14.11 | 14.25 | 7.77 | 14.60 | 11.59 | 12.00 | 5.18 | 26.70 | 12.99 | 11.25 |

**Supplementary Figure S1:** a) and b) Tippers applying sediment over fields; c) and d) Tractors applying sediment over fields; d) Dimensions of tractors loads; e) considered geometry of the top part (also highlighted by green lines on d)). Photo credit: C. Gomez, 2021 (a and b) and 2017 (c and d).


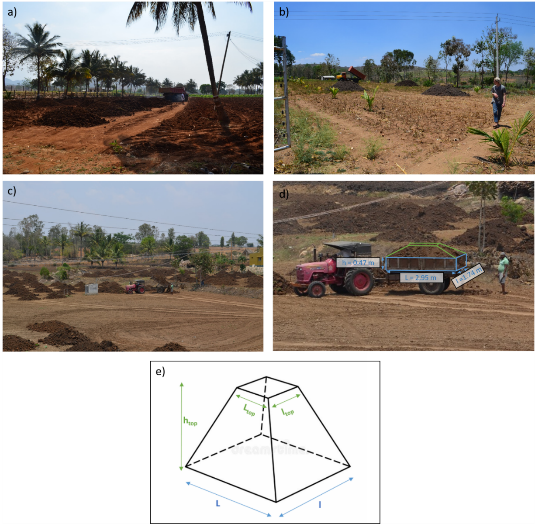


**Supplementary Figure S2**: Distribution of the physico-chemical properties for the different 26 groups of samples: soil before application (N= 8), soil after mixing (N=4) and bottom sediments (N=14). Tukey test results are depicted as lower-case letters (only when the assumptions of the ANOVA were respected, i.e. for clay, silt, sand, CEC, TOC and OM).


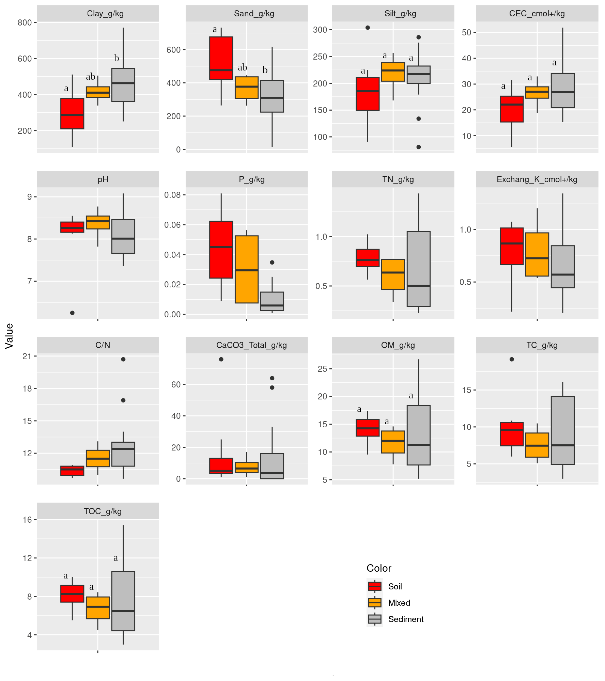

Supplement: Supplementary file 3 — Supplementary Material 3 [file 41598_2025_92206_MOESM3_ESM.docx]
